# Adherence to Guidelines for Adult (Non-GIST) Soft Tissue Sarcoma in the Netherlands: A Plea for Dedicated Sarcoma Centers

**DOI:** 10.1245/s10434-017-6003-3

**Published:** 2017-07-26

**Authors:** Harald J. Hoekstra, Rick L. M. Haas, Cornelis Verhoef, Albert J. H. Suurmeijer, Carla S. P. van Rijswijk, Ben G. H. Bongers, Winette T. van der Graaf, Vincent K. Y. Ho

**Affiliations:** 1 Department of Surgical Oncology, University of Groningen, University Medical Center Groningen, Groningen, The Netherlands; 2 Department of Radiation Oncology, Netherlands Cancer Institute, Amsterdam, The Netherlands; 3 Department of Surgical Oncology, Erasmus MC Cancer Institute, Rotterdam, The Netherlands; 4 Department of Pathology, University of Groningen, University Medical Center Groningen, Groningen, The Netherlands; 5 Department of Radiology, University of Leiden, Leiden University Medical Center, Leiden, The Netherlands; 6 The Institute of Cancer Research and the Royal Marsden NHS Foundation Trust, London, UK; 7 Department of Research, Netherlands Comprehensive Cancer Organization (IKNL), Utrecht, The Netherlands

## Abstract

**Introduction:**

Optimal management of soft tissue sarcoma (STS) remains a challenge. A nationwide survey assessed the quality of STS care in the Netherlands, thereby aiming to identify potentialities for improvement through more centralized disease management.

**Methods:**

From the Netherlands Cancer Registry (NCR), data were obtained on 3317 adult STS patients (excluding gastrointestinal stromal tumor, GIST) diagnosed in 2006–2011. Logistic regression models were employed to compare outcomes on selected clinical indicators reflecting prevailing STS guidelines between high-volume (≥10 resections annually) and low-volume (<10 resections) hospitals, between academic and general hospitals, and between sarcoma research centers and other hospitals, adjusted for case mix. Analyses were performed on imputed datasets (*m* = 50), generated through multiple imputations by chained equations.

**Results:**

Overall, 89% of patients underwent surgical resection. Resection status remained unknown in 24% (excluding those with metastasized disease), and grade was not documented for one-third of tumors. Microscopic residual disease was detected in 20% with an increased risk for older patients, larger and deeply located tumors, and those located in the (retro)peritoneum or upper extremity. Almost half of patients with an R1 resection received adjuvant radiotherapy. Following adjustment for case mix factors, patients treated in high-volume hospitals less often had macroscopic residual disease (R2 resection; adjusted odds ratio: 0.54). A strongly skewed distribution of surgical volumes was observed.

**Conclusions:**

These survey results indicate a potential for improving Dutch STS care. More centralized sarcoma management should improve definitive pathology reporting on tumor characteristics, adherence to treatment guidelines and overall disease outcome.

**Electronic supplementary material:**

The online version of this article (doi:10.1245/s10434-017-6003-3) contains supplementary material, which is available to authorized users.

Soft tissue sarcomas (STS) form a heterogeneous group of tumors deriving from mesenchymal progenitor cells, often showing differentiation towards different mesenchymal cells (e.g., fibrous tissue, adipose tissue, smooth and striated muscle). The etiology remains unknown. They account for 1% of all malignant tumors; incidence rates increase with age and show a slight male predominance.^1^,^2^

STS are primarily defined by the local aggressive growth pattern and categorized in more than 50 histological subtypes.^3^,^4^ Tumor entities vary substantially in biological behavior and tumor grade indicate tendency for distant metastasis, mainly to the lungs.^5^,^6^ Approximately 25% of STS patients develop distant metastases, whereas lymphogenic spread is very rare, affecting only 2–3% of all STS patients.^7^–^9^ Besides STS subtype and grade, studies have identified anatomic site, tumor stage, size, and depth as predictors for survival.^10^ These parameters are included in several nomograms to estimate prognosis regarding local recurrence and survival.^11^ Hence, obtaining accurate information on these factors is crucial for formulating a multidisciplinary treatment plan.

The diverse presentation and localization of STS contribute to the challenges for optimal patient care. Timely diagnosis, for instance, may be difficult given the benign to malignant ratio of soft tissue tumors that has been estimated as 100:1. Furthermore, because of their rarity, physicians seldom encounter STS patients in their practice. Even if recognized as such, the complexities in the diagnostic workup and treatment of STS require adequate expertise and organization.^7^

Studies have emphasized the importance of guideline adherence in STS care as it results in better patient outcomes.^12^^–^ 14 In the Netherlands, national sarcoma guidelines were established in 1993, with updates in 2003 and 2011.^15^ Key recommendations (which became affirmed after our study period through the international guidelines issued by the European Society for Medical Oncology, ESMO) concerned the adequate pathology reporting of the main prognostic sarcoma characteristics, complete diagnostic workup of large (>5 cm), deeply located STS (imaging supplemented with pathologic examination) and the use of adjuvant radiotherapy after R1 resection (microscopic residual disease) or tumor spill.^16^^–^ 18

The purpose of this nationwide survey was to acquire insight of the performance of hospitals on STS care (excluding gastrointestinal stromal tumor, GIST), thereby identifying reference points for furthering quality of sarcoma care. In addition the potential improvement due to centralization of STS care was explored by comparing performance between high- and low-volume hospitals, academic centers versus general hospitals, and sarcoma research centers versus other hospitals.

## Patients and Methods

### Database

The Netherlands Cancer Registry (NCR), founded in 1989, includes all newly diagnosed malignancies, currently covering 17 million inhabitants. The main source of notification is the nationwide network and registry of histo- and cytopathology (www.palga.nl) and case ascertainment was achieved by linkage with the central hospital discharge registry. Upon notification, registrars gather data on patient and tumor characteristics and primary treatment modalities by extracting information directly from the hospital files. The NCR reports on national cancer incidence, prevalence, survival and mortality (www.cijfersoverkanker.nl). Consent for the design, data abstraction process, as well as storage protocols for this study was obtained from the supervisory committee of the NCR.

NCR data include patients’ age at diagnosis, histological subtype of sarcoma on the basis of the World Health Organization (WHO) classification 2002, and tumor grade according to the grading system of the French Federation of Cancer Centers (FNCLCC) Sarcoma Group.^4^,^19^ Primary sites are translated to ICD-O topography codes and tumor stage (depth and size) is recorded according to the TNM system of the International Union Against Cancer (UICC) supplemented with the Extent of Disease code of the American Surveillance, Epidemiology and End Results (SEER) program if available.^20^^–^ 23 Therapy is coded in sequence of administration, with codes differentiating between treatment modalities (surgery, radiotherapy, systemic therapy). In case of surgical treatment, date of resection and residual disease status are recorded.

For this study, data were retrieved on adult STS patients (≥18 years) diagnosed during the period 2006–2011, excluding Kaposi sarcoma, GIST, and sarcoma of the skin (Supplementary Table 1). Hospitals performing sarcoma surgery were classified according to their mean annual number of resections over the total study period as either high-volume (≥10 resections) or low-volume (<10 resections) and in addition hospital type (general vs. academic hospitals) and sarcoma research centers versus other hospitals. Research centers were characterized by participation in the European Organization for Research and Treatment of Cancer (EORTC) sarcoma group, which amongst others implies expertise of dedicated multidisciplinary sarcoma teams and centralized pathology review. These centers are Netherlands Cancer Institute Amsterdam, University Medical Center Rotterdam, University Medical Center Groningen, University Medical Center Nijmegen, and University Medical Center Leiden.

### Clinical Indicators

Guideline adherence was evaluated with indicators reflecting quality of STS care and for which data were available in the NCR. The quality of pathology reports was determined by the availability of information on sarcoma subtype, grade, and assessment of residual disease status after surgery.^24^ In assessing the reports on grading, we excluded sarcoma subtypes that are not graded by definition or for which grading was not recommended: MPNST, angiosarcoma, extraskeletal myxoid chondrosarcoma, alveolar soft part sarcoma, clear cell sarcoma, and epithelioid sarcoma.^19^ Histomorphological codes M8800–M8806 were considered sarcoma lacking specific subtyping, and the availability of grade was assessed separately in the subgroup of liposarcoma (excluding well-differentiated tumors), fibrosarcoma, and leiomyosarcoma. In rating adequate reporting of residual disease, M1 disease and retroperitoneal tumors were excluded from the analyses.

The quality of the diagnostic workup of STS was evaluated by estimating the proportion of possible “whoops” resections. Although the NCR data did not distinguish between planned and unplanned procedures, potentially unplanned procedures were defined as resections of either large (>5 cm) or deep tumors (located beneath the superficial fascia, or with invasion of or through the fascia) without prior histopathologic information. In these cases, the date of first histopathologic confirmation coincided with the date of surgery.

In evaluating the use of adjuvant therapy, analysis was focused on the proportion of patients receiving radiation therapy. In particular, the prevalent guidelines recommend provision of adjuvant radiotherapy to patients with R1 resections, irrespective of their tumors’ grade. For the evaluation of adjuvant treatment, cases with distant disease and retroperitoneal tumors were excluded. In addition, overall 5-year survival rates for high-grade, nonmetastasized tumors in surgically resected patients were estimated.

### Statistical Analyses

Resection rates and rates of R1 resections were tabulated by subgroups of patient and STS characteristics, and differences for significance were tested by *χ* ^2^ tests. Potential prognostic factors for R1 resections were selected for evaluation in multivariable logistic regression on the basis of *p* < 0.1 in the univariable analyses. Odds ratios were calculated together with 95% likelihood ratio confidence intervals.

Performances on most clinical indicators were analyzed as binary variables and logistic regressions were again applied to estimate the impact of hospital types on management of STS patients. Overall survival rates and hazard ratios were estimated in proportional hazards models. To account for missing data, multiple imputations were performed by chained equations under the assumption of missingness being random, thereby creating 50 data sets for each estimation. In addition to crude pooled estimates, we also provided odds and hazard ratios based on the imputed data adjusted for relevant case mix factors. All tests were two-sided and *p* < 0.05 was considered statistically significant. Statistical analyses were performed using Stata (version 14.0; StataCorp, College Station, TX).

## Results

### Patient and Tumor Characteristics

In total 3317 patients, 1830 men (55%) and 1487 women (45%), were diagnosed with a primary STS (Table 1). The median age was 63 (interquartile range 50–75) years; 47% was >65 years. Tumors were mostly located in the extremities (47%), e.g., lower extremity (34%) and upper extremity (13%), followed by the trunk (36%), head and neck region (11%), and retroperitoneum (7%). The majority of tumors were high-grade (54%). In more than a quarter of cases (28%), no information on grade could be retrieved from the pathology report. Almost one-third of STS (32%) was considered small (<5 cm), 61% larger than 5 cm, and in 7% the tumor size could not be retrieved from the clinical or pathology report. Superficially and deeply located tumors were approximately evenly distributed (46 and 43%, respectively), whereas depth was not reported in 12%. Metastatic disease was encountered in 14%. Liposarcoma (20%) and leiomyosarcoma (21%) comprised the most prevalent histology (Table 2).

**Table 1:** General characteristics and resection rates for adult patients (≥18 years) diagnosed with a soft tissue sarcoma (STS) in the Netherlands during the time period 2006–2011

	Total (*n* = 3317)		Resection (*n* = 2698; 81.3%)			R1 resection (*n* = 393; 20.4%)*		
	*n*	%	*n*	%	*p*	*n*	%	*p*
Sex					0.10			0.20
Male	1830	55.1%	1507	55.9%		205	52.2%	
Female	1487	44.9%	1191	44.1%		188	47.8%	
Age at diagnosis (year)					<0.00			<0.00
18–49	794	23.9%	691	25.6%		85	21.6%	
50–64	965	29.1%	807	29.9%		99	25.2%	
65–79	1036	31.2%	823	30.5%		130	33.1%	
≥80	522	15.7%	377	14.0%		79	20.1%	
Median (interquartile range)	63 (50–75)		62 (49–74)			66 (52–77)		
Primary tumor site					<0.00			<0.00
Head and neck	354	10.7%	293	10.9%		44	11.2%	
Trunk	1192	35.9%	856	31.7%		98	24.9%	
(Retro)peritoneum	227	6.8%	169	6.3%		39	9.9%	
Extremity	1544	46.6%	1380	51.2%		212	53.9%	
Upper	431	13.0%	392	14.5%		75	19.1%	
Lower	1113	33.6%	988	36.6%		137	34.9%	
Tumor grade					<0.00			0.95
Low grade	566	17.1%	528	19.6%		76	19.3%	
High grade	1838	55.4%	1526	56.6%		246	62.6%	
Unknown	913	27.5%	644	23.9%		71	18.1%	
Tumor size**					<0.00			<0.00
≤5 cm	933	32.4%	892	38.2%		120	30.5%	
>5 cm	1752	60.9%	1334	57.2%		262	66.7%	
Unknown	193	6.7%	107	4.6%		11	2.8%	
Depth of tumor**					<0.00			0.01
Superficial	1437	45.7%	1355	52.5%		179	45.6%	
Deep	1348	42.9%	996	38.6%		178	45.3%	
Unknown	360	11.5%	232	9.0%		36	9.2%	
Stage					<0.00			–
Localized disease	2838	85.6%	2525	93.6%		393	100.0%	
Distant metastases	479	14.4%	173	6.4%		–	–	

* Excluding R2 resections, cases for which residual disease could not be determined (RX) and metastatic disease

** Excluding cases for which extent of disease (EOD) stage could be determine

**Table 2:** STS subtypes diagnosed in adult patients (≥18 years) in the Netherlands during the time period 2006–2011

Sarcoma subtype (WHO 2002)	Total		Median age (interquartile range)	Male/female
	*n*	%	Year	%/%
Liposarcoma	668	20.1	60 (49–71)	59.6/40.4
Well-differentiated liposarcoma	256	7.7	61 (53–71)	58.6/41.4
Myxoid liposarcoma	158	4.8	46 (37–60)	57.0/43.0
Round cell liposarcoma	10	0.3	47 (44–52)	90.0/10.0
Pleomorphic liposarcoma	48	1.4	67 (60–76)	58.3/41.7
Dedifferentiated liposarcoma	150	4.5	65 (56–73)	59.3/40.7
Mixed-type liposarcoma	8	0.2	65 (48–74)	75.0/25.0
Liposarcoma nos	38	1.1	70 (57–79)	68.4/31.6
Fibrosarcoma	381	11.5	65 (55–76)	55.6/44.4
Well-differentiated fibrosarcoma	83	2.5	60 (48–74)	45.8/54.2
Conventional fibrosarcoma	66	2.0	65 (57–77)	56.1/43.9
Poorly differentiated fibrosarcoma	135	4.1	68 (61–80)	56.3/43.7
Fibrosarcoma nos	97	2.9	64 (48–75)	62.9/37.1
Leiomyosarcoma	701	21.1	64 (53–75)	56.5/43.5
Well-differentiated leiomyosarcoma	148	4.5	61 (50–73)	58.8/41.2
Conventional leiomyosarcoma	180	5.4	64 (53–75)	55.0/45.0
Poorly differentiated/plesiomorphic/epithelioid leiomyosarcoma	137	4.1	64 (57–75)	51.8/48.2
Leiomyosarcoma nos	236	7.1	67 (54–76)	58.9/41.1
Rhabdomyosarcoma	91	2.7	56 (42–65)	67.0/33.0
(Embryonal) rhabdomyosarcoma	40	1.2	53 (35–64)	75.0/25.0
Alveolar rhabdomyosarcoma	13	0.4	26 (21–42)	53.8/46.2
Pleomorphic rhabdomyosarcoma	38	1.1	63 (54–71)	63.2/36.8
Epithelioid hemangioendothelioma	15	0.5	48 (36–59)	40.0/60.0
Angiosarcoma	199	6.0	68 (61–79)	33.2/66.8
Synovial sarcoma	111	3.3	45 (32–59)	55.0/45.0
Malignant peripheral nerve sheath tumor (MPNST)	165	5.0	50 (36–67)	55.8/44.2
Malignant fibrous histiocytoma (MFH)/pleomorphic undifferentiated sarcoma (PUS)	338	10.2	71 (60–81)	54.7/45.3
Other sarcoma	648	19.5	64 (49–76)	54.5/45.5
Total	3317	100.0	63 (50–75)	55.2/44.8

Most patients underwent a resection (81%). Patients who did not underwent surgery were relatively older (median age 69 vs. 62 years), more often a tumor in the trunk (54 vs. 32%), and less often in the extremities (26 vs. 51%). Overall, resected tumors exhibited more favorable characteristics: lower grade, relatively smaller, more often superficially located and mostly localized disease. Surgery was performed in 14% of initial stage IV disease. Forty percent of the 1081 operated patients received radiation therapy, whereas 191 patients received chemotherapy (7%) (data not shown).

One-fifth of the resections were R1 resections (20%), thereby excluding cases with macroscopic and unknown residual disease and those primarily diagnosed with distant disease. R1 resections occurred more often in elderly patients and with tumors located in the (retro)peritoneum or upper extremity and less often in the trunk and lower extremity. In addition, surgery for larger STS and deeply located tumors showed an elevated risk of positive resection margins. Among patients who had an R1 resection, the proportion receiving adjuvant radiotherapy was 47%.

### Hospital Characteristics

**Fig. 1:**
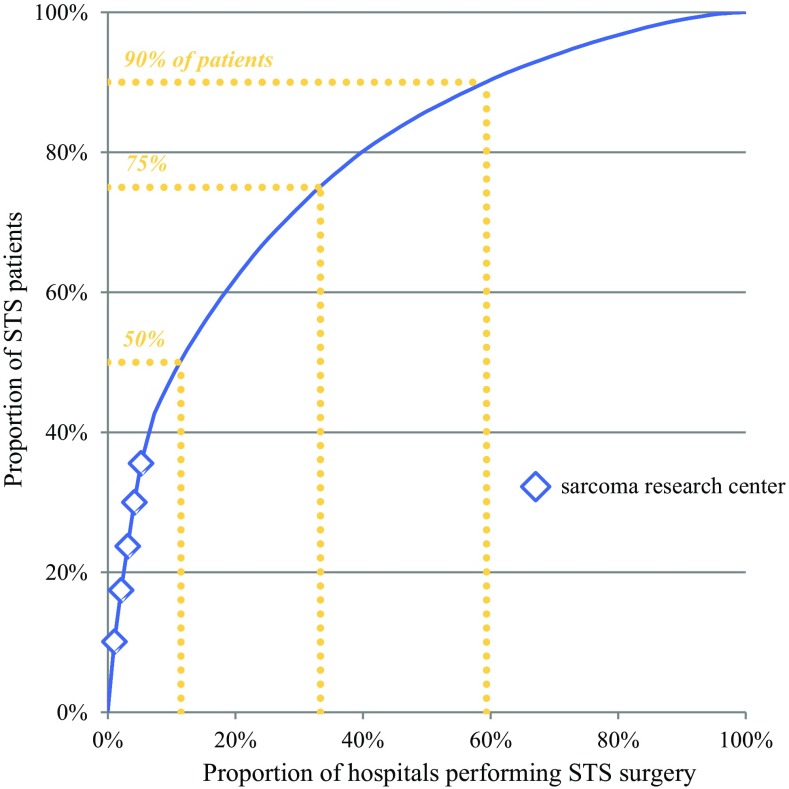
Distribution of patients over hospitals performing STS surgery during the time period 2006–2011

During the study period, diagnostic work and resection of STS was performed in 96 hospitals. Among these were eight academic hospitals and one cancer center (classified in the analyses as academic hospital); five of these were considered sarcoma research centers. A strongly skewed distribution of case volumes by hospital was observed (Fig. 1). Overall, 12% of hospitals accounted for half of all STS resections; this proportion decreased from 16% in 2006 to 10% in 2011. Furthermore, 75% of resections were performed in one-third of hospitals, whereas 90% were performed in almost two-thirds. Sarcoma research centers represented the largest surgical volume.

**Fig. 2:**
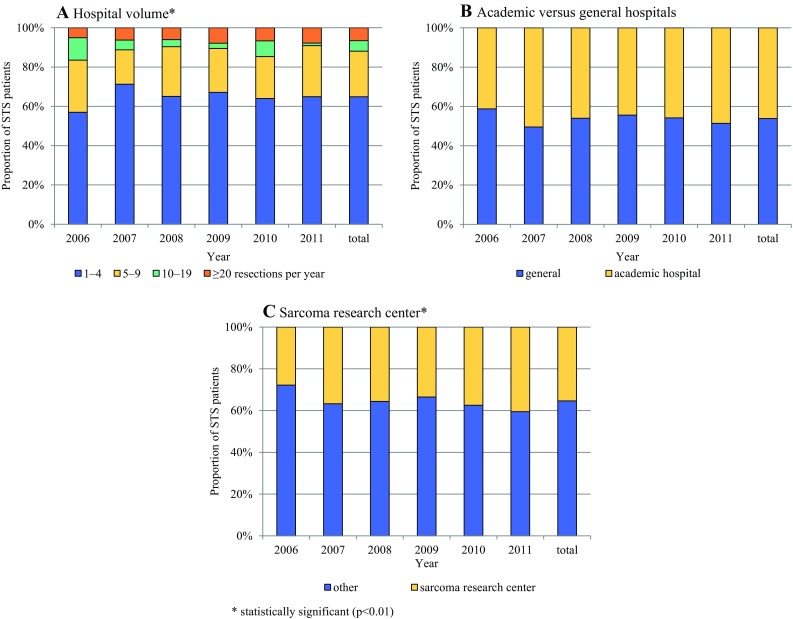
Trends over time according to hospital volume and hospital type with respect to STS surgery

Over time, no trend was detected for whether or not patients underwent surgery in high-volume hospitals. However, we did observe a significant trend within the high-volume group. While the proportion of patients treated in hospitals performing a yearly total of 10–19 resections decreased from 27% in 2006 to 2% in 2011, there was an increase for those treated in hospitals performing 20 or more resections annually (*p* = 0.01; Fig. 2a). Academic hospitals accounted for 46% of STS resections, and this proportion did not show a trend over time (Fig. 2b). In contrast, the proportion of patients treated in sarcoma research centers increased from 28 to 41% (*p* < 0.01; Fig. 2c).

### Clinical Indicators

With respect to pathology reports, sarcoma subtype remained unknown in 20% of patients, tumor grade in 28% (when restricted to the subset of liposarcoma, fibrosarcoma, and leiomyosarcoma cases: 24%) and resection status in 24% (Table 3). High-volume pathology laboratories (compared with low-volume) and those located in academic hospitals (compared to those in general hospitals) and sarcoma research centers (compared with those in other hospitals) performed better in reporting tumor grade, resection status, whereas no difference was observed for reporting sarcoma subtype.

**Table 3:** Variation in clinical indicators by hospital volume and hospital type, with estimations based on imputed data and adjusted for case mix factors (patients’ age, primary tumor site, sarcoma grade, size, and depth, and resection status if relevant)

	Overall	Hospital volume (≥10 cases vs. <10 cases)			Hospital type (academic vs. general)			Sarcoma research center (yes vs. no)		
		Crude	Adjusted		Crude	Adjusted		Crude	Adjusted	
	%	OR	OR	95% CI	OR	OR	95% CI	OR	OR	95% CI
Pathology report: subtype**										
Unknown subtype	20.4%	1.05	1.08	(0.89–1.32)	0.93	0.99	(0.81–1.21)	1.06	1.16	(0.93–1.45)
Pathology report: grade**										
Unknown grade	27.5%	0.85	0.96	(0.80–1.13)	0.53*	0.65*	(0.53–0.78)	0.54*	0.68*	(0.54–0.84)
Liposarcoma, fibrosarcoma, leiomyosarcoma, excluding well-differentiated liposarcoma	24.0%	0.83	0.93	(0.71–1.21)	0.56*	0.65*	(0.48–0.88)	0.56*	0.65*	(0.46–0.93)
Residual disease following resection										
Unknown resection status**	24.0%	0.87	0.99	(0.81–1.21)	0.56*	0.70*	(0.56–0.87)	0.58*	0.73*	(0.57–0.94)
Microscopic residual disease (R1) excluding M1 and (retro)peritoneal tumors	20.0%	1.23	1.22	(0.95–1.57)	1.41*	1.41*	(1.09–1.82)	1.53*	1.56*	(1.22–2.00)
Macroscopic residual disease (R2) excluding M1 and (retro)peritoneal tumors	3.0%	1.00	0.67	(0.37–1.21)	1.24	0.76	(0.42–1.38)	1.23	0.94	(0.52–1.69)
Possible “whoops” resection										
Resection of deep or large tumors (>5 cm) without prior histopathologic confirmation	32.0%	0.19*	0.19*	(0.15–0.24)	0.18*	0.18*	(0.14–0.23)	0.22*	0.20*	(0.16–0.26)
Radiotherapy excluding M1 and (retro) peritoneal tumors										
No radiotherapy neoadjuvant or adjuvant	58.1%	0.39*	0.56*	(0.47–0.68)	0.36*	0.53*	(0.43–0.65)	0.38*	0.53*	(0.43–0.64)
No adjuvant radiotherapy following R1 resection	52.6%	0.43*	0.50*	(0.31–0.80)	0.46*	0.56*	(0.35–0.90)	0.42*	0.47*	(0.29–0.74)

		Crude	Adjusted***		Crude	Adjusted***		Crude	Adjusted***	
	%	HR	HR	95%CI	HR	HR	95% CI	HR	HR	95%CI
Overall survival surgically treated patients, excluding low-grade and M1 disease										
5-Year postdiagnosis	61.3%	1.12	1.15	(0.99–1.34)	1.15*	1.14	(0.97–1.33)	1.10	1.08	(0.93–1.27)

*CI* confidence interval; *OR* odds ratio

* Statistically significant (*p* < 0.05)

** Comparison is calculated on the level of pathology laboratory

*** Additionally adjusted for adjuvant treatment

Following adjustment for case mix factors, resection rates of deep or large tumors without prior histopathologic confirmation seemed considerably higher in low-volume hospitals, general hospitals, and nonsarcoma research centers. In academic hospitals and sarcoma research centers, a larger proportion of operations comprised R1 resections.

The odds for sarcoma patients to receive radiotherapy appeared higher when surgery was performed in high-volume hospitals, academic hospitals, and sarcoma research centers. The same was true regarding adjuvant radiotherapy following R1 resection, although this effect was no longer significant between academic and general hospitals after adjustment for case mix factors. No differences in 5-year overall survival rates were detected between hospital categories.

## Discussion

This survey indicates a need for improving STS care in the Netherlands, both in acquisition and reporting accurate diagnostic information and providing optimal STS treatment. Important STS tumor characteristics remained unmentioned in one-fifth of the pathology reports. The diagnostic workup of large or deeply located STS was incomplete in almost one-third. More than half of patients did not receive the required adjuvant radiation. This does not preclude, however, that patients and physicians may have opted for active surveillance to monitor the tumor’s biological behavior, provided that salvage surgery could be performed in the event of a recurrence.

For particular indicators (grade, resection status, “whoops” resection, and delivery of radiotherapy), outcomes were better when diagnostic and treatment occurred in high-volume hospitals, academic hospitals, and sarcoma research centers compared with other hospitals. Surgery in high-volume hospitals less often resulted in R2 resections. The introduction of evidence-based national STS guidelines produced little effect in contrast to bone sarcoma guidelines.^25^ Compliance to these guidelines was mostly moderate, particularly in smaller hospitals.^14^ Earlier surveys performed in France and the UK also showed disappointing compliance rates regarding STS guidelines, with overall estimates approximating 50% at best, and rates having remained fairly constant over time. 26 ^–^ 30 Such situations do appear to be successfully ameliorated by regional initiatives, for instance directed at improving the quality of pathology reports.^13^

To be sure, strict conformity to guidelines far from qualifies as optimal STS care. For instance, to preserve functionality of limbs affected by STS, positive margins may be justifiable.^31^ This would explain our counterintuitive finding that R1 resections more often occurred in academic hospitals and in sarcoma research centers: an R1 resection oftentimes represents the only treatment option for locally advanced STS.^32^ In case of residual disease, irrespective of it being microscopic or macroscopic cancer, an attempt to obtain adequate margins through reexcision should be evaluated, and the same holds for administration of adjuvant multimodality therapy. As for the observed omittance of adjuvant radiotherapy following an R1 resection, we could not rule out that wait-and-see policies were in fact pursued in such cases. In addition, treatment may have been withheld for both disease and patient-related factors (performance status, comorbidity).^33^

These considerations point to the limitations of this survey, the lack of information on patients’ performance status and comorbidities, the intent of treatment provided as well as the reasons for treatment omittance. As a consequence, case mix corrections may not have adequately accounted for the full variance of baseline characteristics across different hospital categories. Also, we were not able to conclusively infer from the cancer registry data whether resections without prior histopathologic confirmation indeed concerned unplanned excisions (“whoops” procedures). In addition, because no central pathology review was performed, misdiagnosed cases may have been included in the analyses. Nevertheless, the results seem largely valid for assessing STS care in the Netherlands; the analyses were performed on an unselected sample of Dutch STS patients, and main outcomes are consistent with other reports.

In describing the prospects for improving STS care, several challenges remain important to emphasize. Most importantly, with an incidence rate of approximately 3 cases per 100,000 inhabitants (European Standardized Rate), STS represent a group of uncommon tumors that show diverse presentations.^1^ A general practitioner in the Netherlands encounters on average one STS patient every twenty years and a general or orthopedic surgeon one in every four years. At the same time, as mentioned, benign soft-tissue tumors are more than 100 times as common as STS.^34^, ^35^ Misdiagnosis and inadequate treatment are likely to occur.

It is clear that optimal STS care requires extensive, multidisciplinary expertise, and well-organized care processes, and studies have confirmed benefit for management in sarcoma centers, or in hospitals working within specialized, dedicated STS networks.^36^^–^ 39

All in all, improvements in STS care may be achieved by having management primarily carried out in reference centers for sarcomas. Within reference networks, centers may share their multidisciplinary expertise and treat large numbers of patients. In this setting, centralized referral should be pursued as early as possible, preferably at the time of the clinical diagnosis of a suspected sarcoma. In practice, referral of all patients with a lesion likely to be a sarcoma would be recommended. This would mean referring all patients with an unexplained deep mass of soft tissues, or with a superficial lesion of soft tissues having a diameter of >5 cm.^17^ Although more centralized management may come with the cost of large numbers of patients being redirected to centers for benign abnormalities, potentially causing delayed treatment of those with proven STS, some have reported encouraging outcomes for more stringent referral patterns.^40^,^41^ In the Netherlands, national referral guidelines are already well-established for bone sarcoma.

While specific criteria for reference centers may vary from country to country, centralizing STS care should be based, among others, on the availability of state-of-the-art facilities for STS diagnostics and treatment, multidisciplinary expertise (employed in weekly tumor boards discussing new patients, for instance), and larger volumes of patients. Quality of STS care delivered should be monitored and outcomes reported on a regular basis. Also, centers are involved in ongoing clinical trials, for which patients’ enrollment is common.

In the Netherlands, centralizing STS care in five dedicated sarcoma centers, in analogy with the four national bone sarcoma centers, may enhance the development and implementation of new diagnostics (e.g., imaging technologies and improved STS subtyping according to tumors’ molecular makeup) and therapeutic strategies (introduction of new combined, pathway driven treatment modalities). Moreover, restricting the number of centers should facilitate a nationwide pathology review and reporting standard, and foster focused research initiatives on the mentioned themes. Hence, centralization may prove most beneficial for establishing disease-orientated sarcoma care, including participation in trials, which eventually should lead not only to more favorable clinical outcomes, but also to more efficient and cost-effective STS.^42^ It is time for integrated sarcoma care, in the Netherlands and in Europe.^43^

## Electronic supplementary material

Below is the link to the electronic supplementary material.

Supplementary material 1 (DOCX 88 kb)
