# Supplementary material for: Adherence to Guidelines for Adult (Non-GIST) Soft Tissue Sarcoma in the Netherlands: A Plea for Dedicated Sarcoma Centers
Source: Ann Surg Oncol. 2017 Jul 26;24(11):3279–88. doi: 10.1245/s10434-017-6003-3 (PMC5596052; doi:10.1245/s10434-017-6003-3)
Supplement: Supplementary file 1 — Supplementary material 1 (DOCX 88 kb) [file 10434_2017_6003_MOESM1_ESM.docx]

**Supplementary table 1**

Classification of STS based on the International Classification of Diseases for Oncology (ICD-O-3) and the World Health Organization (WHO) classification 2002

| **Sarcoma subtype (WHO 2002)** | **Morphology code** | **Grade** |
| --- | --- | --- |
| liposarcoma | M8850–M8858 |  |
| well differentiated liposarcoma | M8851 | low |
| myxoid liposarcoma | M8852 | low |
| round cell liposarcoma | M8853 | high |
| pleomorphic liposarcoma | M8854 | high |
| dedifferentiated liposarcoma | M8858 | high |
| mixed-type liposarcoma | M8855 | high |
| liposarcoma nos | M8850 | unspecified |
| fibrosarcoma | M8810–M8825, M9150 |  |
| well differentiated fibrosarcoma | M8810–M8825, M9150 | low |
| conventional fibrosarcoma | M8810–M8825, M9150 | high |
| poorly differentiated fibrosarcoma | M8810–M8825, M9150 | high |
| fibrosarcoma nos | M8810–M8825, M9150 | unspecified |
| leiomyosarcoma | M8890–M8896 |  |
| well differentiated leiomyosarcoma | M8890–M8896 | low |
| conventional leiomyosarcoma | M8890–M8896 | high |
| poorly differentiated leiomyosarcoma | M8890–M8896 | high |
| leiomyosarcoma nos | M8890–M8896 | unspecified |
| rhabdomyosarcoma | M8895, M8900–M8902, M8910–M8912, M8920–M8921, M8991 |  |
| (embryonal) rhabdomyosarcoma | M8895, M8900–M8902, M8910–M8912, M8991 | high |
| alveolar rhabdomyosarcoma | M8902, M8920–M8921 | high |
| pleomorphic rhabdomyosarcoma | M8901 | high |
| epithelioid haemangioendothelioma | M9130, M9133 | low–high |
| angiosarcoma | M9120, M9170 | high** |
| synovial sarcoma | M9040–M 9043 | high |
| MPNST | M9540–9571 | high** |
| MFH/undifferentiated pleiomorphic sarcoma | M8830–M8831, M9251–M9252 | high |
| other sarcoma types | various* | various |

* These include: glomus tumour (M8711), PNET/extraskeletal Ewing tumour (M9260, M9364, M9365), clear cell sarcoma of soft tissue (M9044), extra-renal rhabdoid tumour (M8963) and sarcoma nos, including malignant mesenchymoma (M8990).

** Grades were assigned according to the WHO classification 2013
